# 2,6-Dimethyl-4-*m*-tolyl­cyclo­hex-3-enecarboxylic acid

**DOI:** 10.1107/S1600536808027542

**Published:** 2008-09-06

**Authors:** Songwen Xie, Holly J. Stein, Maren Pink

**Affiliations:** a Department of Natural Information and Mathematical Sciences, Indiana University Kokomo, Kokomo, IN 46904-9003, USA; b Indiana University Molecular Structure Center, Indiana University, Bloomington, IN 47405-7102, USA

## Abstract

The title compound, C_16_H_20_O_2_, was synthesized to study the hydrogen-bonding inter­action of the two enanti­omers in the solid state. The racemate is made up of carboxylic acid *RS* dimers. Inter­molecular O—H⋯O hydrogen bonds produce centrosymmetric *R*_2_^2^(8) rings which dimerize the two chiral enanti­omers through their carboxyl groups. The chirality of this compound is generated by the presence of the double bond in the cyclo­hexene ring and a chiral axis due to the *meta*-methyl substituent on the aromatic ring.

## Related literature

In similar compounds previously reported (Xie *et al.*, 2002 ▶, 2007*a* ▶, 2008 ▶), the racemates also consist of carboxylic acid *RS* dimers. For related literature, see: Xie *et al.* (2007*b* ▶, 2004 ▶); Bernstein *et al.* (1995 ▶).
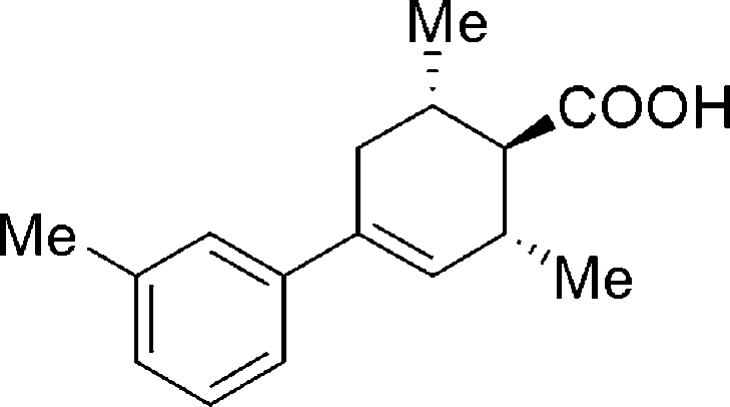


## Experimental

### 

#### Crystal data

C_16_H_20_O_2_
*M* *_r_* = 244.32
Orthorhombic, 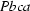

*a* = 11.2581 (10) Å
*b* = 8.1055 (7) Å
*c* = 29.857 (3) Å
*V* = 2724.5 (4) Å^3^
*Z* = 8
Mo *K*α radiation
μ = 0.08 mm^−1^
*T* = 150 (2) K
0.20 × 0.18 × 0.05 mm

#### Data collection

Bruker Kappa APEXII diffractometer
Absorption correction: multi-scan (*SADABS*; Sheldrick, 1996 ▶) *T*_min_ = 0.985, *T*_max_ = 0.996
18515 measured reflections
3120 independent reflections
2265 reflections with *I* > 2σ(*I*)
*R*_int_ = 0.043

#### Refinement

*R*[*F* ^2^ > 2σ(*F* ^2^)] = 0.049
*wR*(*F* ^2^) = 0.136
*S* = 1.03
3120 reflections
170 parameters
H atoms treated by a mixture of independent and constrained refinement
Δρ_max_ = 0.50 e Å^−3^
Δρ_min_ = −0.20 e Å^−3^

### 

Data collection: *APEX2* (Bruker, 2007 ▶); cell refinement: *SAINT* (Bruker, 2007 ▶); data reduction: *SAINT*; program(s) used to solve structure: *SIR2004* (Burla *et al.*, 2005 ▶); program(s) used to refine structure: *SHELXL97* (Sheldrick, 2008 ▶); molecular graphics: *SHELXTL* (Sheldrick, 2008 ▶); software used to prepare material for publication: *SHELXTL*.

## Supplementary Material

Crystal structure: contains datablocks global, I. DOI: 10.1107/S1600536808027542/om2254sup1.cif

Structure factors: contains datablocks I. DOI: 10.1107/S1600536808027542/om2254Isup2.hkl

Additional supplementary materials:  crystallographic information; 3D view; checkCIF report

## Figures and Tables

**Table 1:** Hydrogen-bond geometry (Å, °)

*D*—H⋯*A*	*D*—H	H⋯*A*	*D*⋯*A*	*D*—H⋯*A*
O1—H1*O*⋯O2^i^	1.05 (3)	1.62 (3)	2.6628 (18)	174 (3)

Symmetry code: (i) 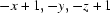.

**Fig. 1.**
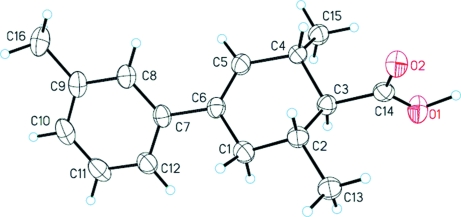
The molecular structure and atom numbering scheme.

**Fig. 2.**
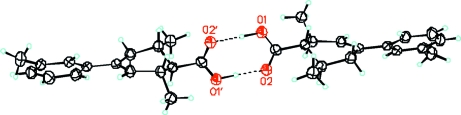
Hydrogen bonded dimer. Dashed lines represent hydrogen bonds. [Symmetry code: #1 -x + 1, -y, -z + 1.]

## supplementary crystallographic information

### Comment

The title carboxylic acid was prepared to study the interaction of the two enantiomers in the solid state. We have previously reported the structure of its precursor, which is achiral and forms hydrogen-bonded dimers (Xie *et al.*, 2007*b*). The chirality of the title compound is generated by the presence of the double bond in the cyclohexene ring (Xie *et al.*, 2004). The resultant racemate is made up of carboxylic acid *RS* dimers (Xie *et al.*, 2002, 2007*a*, 2008). The structure and atom numbering are shown in Fig. 1, which illustrates the half-chair conformation of the cyclohexene ring. The torsion angles involving atoms C4, C5, C6, C1, and C2 are all near 180°, as are those involving atoms C13, C2, C3, C4, and C15. The carboxyl group is almost perpendicular to the cyclohexene ring with an angle of 86.5° between the O1—C14—O2 plane and the C1—C6 ring. The double bond between C5—C6 is not fully conjugated with the aromatic ring as shown by the C1—C6—C5 plane to benzene ring angle of 42.4°. Unlike other previously reported *para* substituted analogs, the molecule also has a chiral axis due to the *meta* methyl substituent on the aromatic ring.

Fig. 2 shows the hydrogen bonding scheme. Atom O1 acts as a donor in an intermolecular hydrogen bond to atom O2, producing an R_2_^2^(8) ring (Bernstein *et al.*, 1995), thus creating a hydrogen-bonded dimer. There is no evidence to suggest that weak directional interactions interconnect the dimers. Hydrogen bond geometry is given in Table 1.

### Experimental

The title carboxylic acid was synthesized following the similar method reported by Xie *et al.*, 2002. The purified compound was recrystallized from hexane-dichloromethane as colorless plates (m.p. 412–415 K).

### Refinement

The hydrogen atoms not involved in hydrogen bonding were placed in idealized positions and refined as riding atoms with relative isotropic displacement parameters; they were positioned geometrically and refined using a riding model with C—H = 0.95 Å and *U*_iso_(H) = 1.2 times *U*_eq_(C). H1O was refined freely with individual displacement parameters.

### Crystal data

**Table:**

C_16_H_20_O_2_	*F*(000) = 1056
*M**_r_* = 244.32	*D*_x_ = 1.191 Mg m^−^^3^
Orthorhombic, *P**b**c**a*	Mo *K*α radiation, λ = 0.71073 Å
Hall symbol: -P 2ac 2ab	Cell parameters from 3951 reflections
*a* = 11.2581 (10) Å	θ = 3.2–25.9°
*b* = 8.1055 (7) Å	µ = 0.08 mm^−^^1^
*c* = 29.857 (3) Å	*T* = 150 K
*V* = 2724.5 (4) Å^3^	Plate, colorless
*Z* = 8	0.20 × 0.18 × 0.05 mm

### Data collection

**Table:**

Bruker Kappa APEXII diffractometer	3120 independent reflections
Radiation source: fine-focus sealed tube	2265 reflections with *I* > 2σ(*I*)
graphite	*R*_int_ = 0.043
Detector resolution: 8.3 pixels mm^-1^	θ_max_ = 27.5°, θ_min_ = 1.4°
ω and φ scans	*h* = −14→12
Absorption correction: multi-scan (SADABS; Sheldrick, 1996)	*k* = −10→10
*T*_min_ = 0.985, *T*_max_ = 0.996	*l* = −38→36
18515 measured reflections	

### Refinement

**Table:**

Refinement on *F*^2^	Primary atom site location: structure-invariant direct methods
Least-squares matrix: full	Secondary atom site location: difference Fourier map
*R*[*F*^2^ > 2σ(*F*^2^)] = 0.049	Hydrogen site location: inferred from neighbouring sites
*wR*(*F*^2^) = 0.136	H atoms treated by a mixture of independent and constrained refinement
*S* = 1.03	*w* = 1/[σ^2^(*F*_o_^2^) + (0.066*P*)^2^ + 0.9686*P*] where *P* = (*F*_o_^2^ + 2*F*_c_^2^)/3
3120 reflections	(Δ/σ)_max_ < 0.001
170 parameters	Δρ_max_ = 0.50 e Å^−^^3^
0 restraints	Δρ_min_ = −0.20 e Å^−^^3^

### Special details

**Table:**

Geometry. All e.s.d.'s (except the e.s.d. in the dihedral angle between two l.s. planes) are estimated using the full covariance matrix. The cell e.s.d.'s are taken into account individually in the estimation of e.s.d.'s in distances, angles and torsion angles; correlations between e.s.d.'s in cell parameters are only used when they are defined by crystal symmetry. An approximate (isotropic) treatment of cell e.s.d.'s is used for estimating e.s.d.'s involving l.s. planes.
Refinement. Refinement of *F*^2^ against ALL reflections. The weighted *R*-factor *wR* and goodness of fit *S* are based on *F*^2^, conventional *R*-factors *R* are based on *F*, with *F* set to zero for negative *F*^2^. The threshold expression of *F*^2^ > σ(*F*^2^) is used only for calculating *R*-factors(gt) *etc*. and is not relevant to the choice of reflections for refinement. *R*-factors based on *F*^2^ are statistically about twice as large as those based on *F*, and *R*- factors based on ALL data will be even larger.

### Fractional atomic coordinates and isotropic or equivalent isotropic displacement parameters (Å^2^)

**Table:**

	*x*	*y*	*z*	*U*_iso_*/*U*_eq_	
O1	0.47404 (12)	0.21830 (16)	0.50798 (4)	0.0403 (3)	
H1O	0.477 (3)	0.103 (4)	0.5238 (11)	0.113 (11)*	
O2	0.51225 (11)	0.06571 (15)	0.44750 (4)	0.0362 (3)	
C1	0.56818 (15)	0.5618 (2)	0.38758 (6)	0.0333 (4)	
H1A	0.5723	0.6504	0.4104	0.040*	
H1B	0.6339	0.5799	0.3661	0.040*	
C2	0.58687 (14)	0.3953 (2)	0.41086 (6)	0.0308 (4)	
H2	0.5978	0.3087	0.3873	0.037*	
C3	0.47402 (14)	0.3553 (2)	0.43738 (5)	0.0274 (4)	
H3	0.4578	0.4494	0.4582	0.033*	
C4	0.36497 (13)	0.3349 (2)	0.40608 (5)	0.0272 (3)	
H4	0.3711	0.2262	0.3904	0.033*	
C5	0.36254 (14)	0.4705 (2)	0.37130 (6)	0.0309 (4)	
H5	0.2923	0.4822	0.3540	0.037*	
C6	0.45258 (14)	0.57605 (19)	0.36300 (5)	0.0262 (3)	
C7	0.43895 (13)	0.7170 (2)	0.33124 (5)	0.0282 (4)	
C8	0.37328 (15)	0.7010 (2)	0.29151 (5)	0.0316 (4)	
H8	0.3374	0.5979	0.2846	0.038*	
C9	0.35932 (15)	0.8328 (2)	0.26180 (6)	0.0354 (4)	
C10	0.41173 (16)	0.9834 (2)	0.27251 (7)	0.0413 (5)	
H10	0.4038	1.0740	0.2525	0.050*	
C11	0.47524 (17)	1.0027 (2)	0.31192 (7)	0.0421 (5)	
H11	0.5088	1.1070	0.3191	0.051*	
C12	0.49008 (15)	0.8706 (2)	0.34103 (6)	0.0351 (4)	
H12	0.5351	0.8843	0.3677	0.042*	
C13	0.69806 (14)	0.4001 (2)	0.43992 (6)	0.0360 (4)	
H13A	0.7662	0.4336	0.4216	0.054*	
H13B	0.7127	0.2903	0.4525	0.054*	
H13C	0.6868	0.4795	0.4643	0.054*	
C14	0.48878 (13)	0.2002 (2)	0.46525 (6)	0.0284 (4)	
C15	0.25044 (15)	0.3372 (2)	0.43369 (6)	0.0333 (4)	
H15A	0.1820	0.3237	0.4137	0.050*	
H15B	0.2440	0.4427	0.4496	0.050*	
H15C	0.2520	0.2468	0.4555	0.050*	
C16	0.2881 (2)	0.8129 (3)	0.21924 (6)	0.0483 (5)	
H16A	0.2877	0.6965	0.2103	0.072*	
H16B	0.3240	0.8793	0.1954	0.072*	
H16C	0.2064	0.8500	0.2244	0.072*	

### Atomic displacement parameters (Å^2^)

**Table:**

	*U*^11^	*U*^22^	*U*^33^	*U*^12^	*U*^13^	*U*^23^
O1	0.0541 (8)	0.0364 (7)	0.0303 (7)	0.0076 (6)	0.0021 (6)	0.0048 (6)
O2	0.0399 (6)	0.0362 (7)	0.0327 (7)	0.0045 (5)	0.0002 (5)	0.0041 (5)
C1	0.0313 (8)	0.0340 (9)	0.0346 (9)	−0.0030 (7)	0.0007 (7)	0.0049 (7)
C2	0.0261 (8)	0.0334 (9)	0.0330 (9)	−0.0012 (6)	−0.0007 (7)	0.0037 (7)
C3	0.0260 (7)	0.0288 (8)	0.0273 (8)	0.0012 (6)	0.0000 (6)	0.0012 (7)
C4	0.0251 (7)	0.0273 (8)	0.0292 (8)	−0.0019 (6)	−0.0039 (6)	0.0038 (7)
C5	0.0287 (8)	0.0330 (9)	0.0310 (9)	0.0013 (6)	−0.0041 (7)	0.0052 (7)
C6	0.0298 (7)	0.0267 (8)	0.0222 (8)	0.0020 (6)	0.0022 (6)	−0.0003 (6)
C7	0.0275 (7)	0.0306 (8)	0.0264 (8)	0.0031 (6)	0.0068 (6)	0.0046 (7)
C8	0.0346 (8)	0.0319 (9)	0.0282 (8)	0.0042 (7)	0.0046 (7)	0.0032 (7)
C9	0.0339 (8)	0.0419 (10)	0.0305 (9)	0.0081 (7)	0.0059 (7)	0.0096 (8)
C10	0.0366 (9)	0.0398 (10)	0.0474 (11)	0.0026 (8)	0.0077 (8)	0.0184 (9)
C11	0.0384 (9)	0.0328 (9)	0.0551 (12)	−0.0057 (8)	0.0049 (9)	0.0107 (9)
C12	0.0329 (8)	0.0356 (9)	0.0368 (9)	−0.0041 (7)	0.0029 (7)	0.0054 (8)
C13	0.0257 (8)	0.0414 (10)	0.0410 (10)	−0.0020 (7)	−0.0027 (7)	0.0061 (8)
C14	0.0199 (7)	0.0289 (8)	0.0365 (9)	0.0000 (6)	−0.0029 (6)	0.0017 (7)
C15	0.0293 (8)	0.0363 (9)	0.0344 (9)	0.0007 (7)	−0.0020 (7)	0.0023 (7)
C16	0.0602 (12)	0.0509 (12)	0.0338 (10)	0.0090 (10)	−0.0029 (9)	0.0125 (9)

### Geometric parameters (Å, °)

**Table:**

O1—C14	1.295 (2)	C7—C8	1.404 (2)
O1—H1O	1.05 (3)	C8—C9	1.398 (2)
O2—C14	1.241 (2)	C8—H8	0.9500
C1—C6	1.499 (2)	C9—C10	1.393 (3)
C1—C2	1.533 (2)	C9—C16	1.511 (3)
C1—H1A	0.9900	C10—C11	1.386 (3)
C1—H1B	0.9900	C10—H10	0.9500
C2—C13	1.524 (2)	C11—C12	1.390 (3)
C2—C3	1.532 (2)	C11—H11	0.9500
C2—H2	1.0000	C12—H12	0.9500
C3—C14	1.517 (2)	C13—H13A	0.9800
C3—C4	1.552 (2)	C13—H13B	0.9800
C3—H3	1.0000	C13—H13C	0.9800
C4—C5	1.512 (2)	C15—H15A	0.9800
C4—C15	1.530 (2)	C15—H15B	0.9800
C4—H4	1.0000	C15—H15C	0.9800
C5—C6	1.349 (2)	C16—H16A	0.9800
C5—H5	0.9500	C16—H16B	0.9800
C6—C7	1.493 (2)	C16—H16C	0.9800
C7—C12	1.402 (2)		
			
C14—O1—H1O	109.7 (18)	C9—C8—H8	119.2
C6—C1—C2	114.13 (13)	C7—C8—H8	119.2
C6—C1—H1A	108.7	C10—C9—C8	118.46 (16)
C2—C1—H1A	108.7	C10—C9—C16	120.77 (16)
C6—C1—H1B	108.7	C8—C9—C16	120.77 (17)
C2—C1—H1B	108.7	C11—C10—C9	120.85 (16)
H1A—C1—H1B	107.6	C11—C10—H10	119.6
C13—C2—C3	113.10 (14)	C9—C10—H10	119.6
C13—C2—C1	110.39 (14)	C10—C11—C12	120.39 (18)
C3—C2—C1	107.85 (13)	C10—C11—H11	119.8
C13—C2—H2	108.5	C12—C11—H11	119.8
C3—C2—H2	108.5	C11—C12—C7	120.30 (17)
C1—C2—H2	108.5	C11—C12—H12	119.9
C14—C3—C2	111.58 (13)	C7—C12—H12	119.9
C14—C3—C4	109.18 (13)	C2—C13—H13A	109.5
C2—C3—C4	111.55 (13)	C2—C13—H13B	109.5
C14—C3—H3	108.1	H13A—C13—H13B	109.5
C2—C3—H3	108.1	C2—C13—H13C	109.5
C4—C3—H3	108.1	H13A—C13—H13C	109.5
C5—C4—C15	110.23 (13)	H13B—C13—H13C	109.5
C5—C4—C3	110.51 (13)	O2—C14—O1	123.21 (15)
C15—C4—C3	109.93 (13)	O2—C14—C3	121.15 (15)
C5—C4—H4	108.7	O1—C14—C3	115.63 (15)
C15—C4—H4	108.7	C4—C15—H15A	109.5
C3—C4—H4	108.7	C4—C15—H15B	109.5
C6—C5—C4	124.97 (14)	H15A—C15—H15B	109.5
C6—C5—H5	117.5	C4—C15—H15C	109.5
C4—C5—H5	117.5	H15A—C15—H15C	109.5
C5—C6—C7	121.65 (14)	H15B—C15—H15C	109.5
C5—C6—C1	120.91 (14)	C9—C16—H16A	109.5
C7—C6—C1	117.33 (13)	C9—C16—H16B	109.5
C12—C7—C8	118.32 (15)	H16A—C16—H16B	109.5
C12—C7—C6	120.32 (15)	C9—C16—H16C	109.5
C8—C7—C6	121.34 (15)	H16A—C16—H16C	109.5
C9—C8—C7	121.65 (16)	H16B—C16—H16C	109.5
			
C6—C1—C2—C13	−172.47 (14)	C1—C6—C7—C12	−35.8 (2)
C6—C1—C2—C3	−48.44 (19)	C5—C6—C7—C8	−38.1 (2)
C13—C2—C3—C14	−52.09 (19)	C1—C6—C7—C8	145.50 (15)
C1—C2—C3—C14	−174.45 (14)	C12—C7—C8—C9	0.7 (2)
C13—C2—C3—C4	−174.51 (14)	C6—C7—C8—C9	179.46 (15)
C1—C2—C3—C4	63.12 (17)	C7—C8—C9—C10	−0.4 (2)
C14—C3—C4—C5	−168.20 (13)	C7—C8—C9—C16	−179.91 (16)
C2—C3—C4—C5	−44.41 (18)	C8—C9—C10—C11	−0.7 (3)
C14—C3—C4—C15	69.91 (17)	C16—C9—C10—C11	178.81 (18)
C2—C3—C4—C15	−166.30 (13)	C9—C10—C11—C12	1.5 (3)
C15—C4—C5—C6	133.15 (17)	C10—C11—C12—C7	−1.2 (3)
C3—C4—C5—C6	11.4 (2)	C8—C7—C12—C11	0.1 (2)
C4—C5—C6—C7	−173.87 (15)	C6—C7—C12—C11	−178.63 (15)
C4—C5—C6—C1	2.4 (3)	C2—C3—C14—O2	−59.64 (19)
C2—C1—C6—C5	17.0 (2)	C4—C3—C14—O2	64.14 (18)
C2—C1—C6—C7	−166.55 (14)	C2—C3—C14—O1	121.08 (15)
C5—C6—C7—C12	140.62 (17)	C4—C3—C14—O1	−115.15 (15)

### Hydrogen-bond geometry (Å, °)

**Table:**

*D*—H···*A*	*D*—H	H···*A*	*D*···*A*	*D*—H···*A*
O1—H1O···O2^i^	1.05 (3)	1.62 (3)	2.6628 (18)	174 (3)

Symmetry codes: (i) −*x*+1, −*y*, −*z*+1.
